# Large-Scale Cortical Dynamics of Sleep Slow Waves

**DOI:** 10.1371/journal.pone.0030757

**Published:** 2012-02-17

**Authors:** Vicente Botella-Soler, Mario Valderrama, Benoît Crépon, Vincent Navarro, Michel Le Van Quyen

**Affiliations:** 1 Departament de Física Teòrica and Instituto de Física Corpuscular (IFIC), Universitat de València - Consejo Superior de Investigaciones Científicas (CSIC), Burjassot, València, Spain; 2 Epilepsy Unit, AP-HP, Hôpital de la Pitié Salpêtrière, Paris, France; 3 Centre de Recherche de l'Institut du Cerveau et de la Moelle épinière (CRICM), Centre National de la Recherche Scientifique (CNRS) UMR 7225, Institut National de la Santé et de la Recherche Médicale (INSERM) UMRS 975, Université Pierre et Marie Curie (UPMC), Hôpital de la Pitié Salpêtrière, Paris, France; Indiana University, United States of America

## Abstract

Slow waves constitute the main signature of sleep in the electroencephalogram (EEG). They reflect alternating periods of neuronal hyperpolarization and depolarization in cortical networks. While recent findings have demonstrated their functional role in shaping and strengthening neuronal networks, a large-scale characterization of these two processes remains elusive in the human brain. In this study, by using simultaneous scalp EEG and intracranial recordings in 10 epileptic subjects, we examined the dynamics of hyperpolarization and depolarization waves over a large extent of the human cortex. We report that both hyperpolarization and depolarization processes can occur with two different characteristic time durations which are consistent across all subjects. For both hyperpolarization and depolarization waves, their average speed over the cortex was estimated to be approximately 1 m/s. Finally, we characterized their propagation pathways by studying the preferential trajectories between most involved intracranial contacts. For both waves, although single events could begin in almost all investigated sites across the entire cortex, we found that the majority of the preferential starting locations were located in frontal regions of the brain while they had a tendency to end in posterior and temporal regions.

## Introduction

Slow waves are the most prominent electroencephalographic (EEG) feature of sleep, consisting of alternating periods of activity and silence in cortical networks [1], [2]. As confirmed by in vivo [3] and in vitro [4] intracellular recordings in animals, the active states are associated with synaptically mediated depolarization and intense cellular firing whereas, during the silence states, neurons are hyperpolarized with a cessation of synaptic and firing activity. This slow rhythmical activity is generated intrinsically in the neocortex [4], [5], synchronizes cortical regions with high temporal precision [6] and can recruit multiple subcortical targets [7], [8]. This alternation of active and silent states in cortical neurons is thought to be involved in memory consolidation [9], [10], [11], synaptic homeostasis [12] and the restorative functions of sleep [13]. Nevertheless, despite considerable understanding of the cellular/synaptic mechanisms underlying these waves [8], [14], the spatial and temporal dynamics that generate these particular EEG elements in the human brain remain poorly specified [15], [16]. Scalp-level high-density EEG recordings have indicated that slow waves in humans have a non-uniform cortical distribution, suggesting that areas of the cortex are differently involved in slow waves [17]. Furthermore, recent observations reported that most sleep slow waves are in fact confined to local regions that are unevenly distributed across cortex [16]. In addition, consistent with recent imaging data of the mouse cortex [18], their propagations are composed of complex local paths with several points of origin [19]. Indubitably, scalp recorded EEG is insufficient to fully characterize their complex large-scale dynamics due to unavoidable effects of linear summations of current sources over large cortical territories and the considerable distances from recording sites to deep generators. These drawbacks can nevertheless be surmounted with the use of intracranial recordings [15], [16] which furthermore allow the analysis of short-range spatially coherent activities that are not promptly available with scalp recordings. In addition, the number of studies simultaneously analyzing local field potential (LFP) and scalp EEG signals both in animals and humans is rather small and therefore joint analyses at mesoscopic (e.g., LFPs) and macroscopic (e.g., scalp EEG) levels will provide valuable new data in this domain. In the present study, we examined slow wave cortical activity during polysomnographically defined sleep-wake states using simultaneous scalp EEG and intracranial recordings in 10 subjects who required a clinical invasive evaluation for the treatment of their epilepsy. Thanks to this relatively large sample size as well as to a broad spatial sampling (with a total of 417 investigated intracranial electrode sites) we were able to examine intracranially the characteristic morphologies, durations and spatial propagations of slow waves over a comprehensive extent of the human cerebral cortex.

## Results

### Bimodal distributions of the depolarization and hyperpolarization times

**Figure 1:**
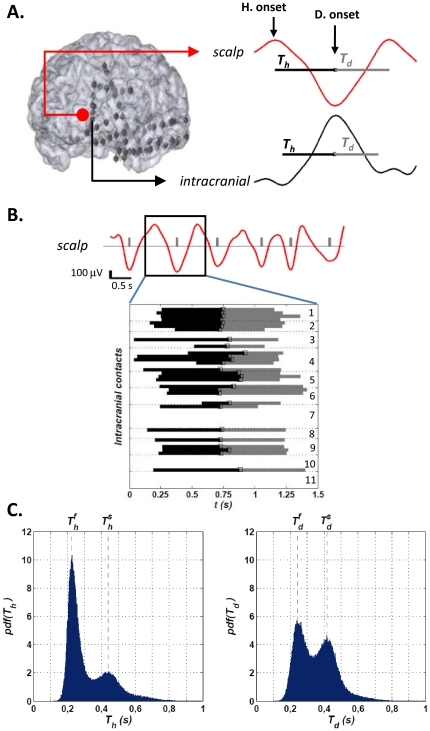
Illustration of the slow wave events detection method and example of T_h_/T_d_ distributions. **A.** *Left*: 3D reconstructed implantation of one subject. The red circle marks the approximate position of the scalp reference contact. *Right*: Example of scalp slow oscillation and one of its intracranial correlates (note the polarity inversion of the intracranial signal with respect to scalp). All signals are filtered in the band 0.1–4.0 Hz. The hyperpolarization and depolarization onsets have been marked for the scalp signal. The duration of the hyperpolarization (T_h_) and depolarization (T_d_) phases of each oscillation is shown as well (black and gray horizontal lines, respectively). **B.** Segment of scalp signal presenting six detected slow waves. For the second detection, the T_h_ and T_d_ of the detected intracranial correlates have been plotted. The numbers stand for contacts of the same electrode. This particular implantation consisted of 55 contacts distributed in 11 electrodes, 6 electrodes (n.1–6, 30 contacts) in the left frontal lobe and 5 (n.7–11, 25 contacts) in the left temporal lobe. The contacts in the frontal lobe cover from the F1, F2 and orbital regions to interior regions like the anterior part of the cingulated gyrus, the insula and the cortex subcallosum. The electrodes in the temporal lobe cover different cortical and white matter regions as well as deeper structures like the amygdala and the hippocampus. **C.** Examples of typical Probability Density Functions of T_h_ and T_d_ obtained for a single subject. For the construction of the pdfs only the intracranially measured T_h_ and T_d_ were taken into account. 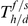 mark the position of the fast/slow hyperpolarization/depolarization times of the subject.

**Figure 2:**
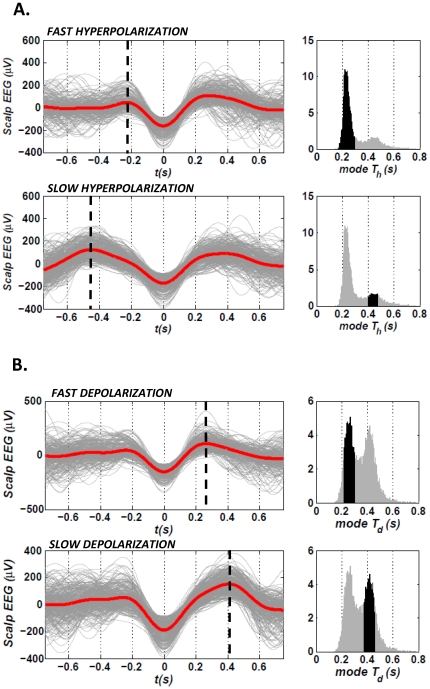
Examples of fast/slow hyperpolarization/depolarization slow waves. **A.** Superposition of filtered signals (0.1–4 Hz) of scalp EEG recordings (FP1) corresponding to 300 slow waves showing fast hyperpolarization (top) and 300 slow waves showing slow hyperpolarization (bottom) of a single subject (S1). The thick red line is the average profile of the shown filtered signals. A vertical dashed line marks the position of the hyperpolarization onset of the average signal. The graphs in the right panels indicate the region in the *mode T_h_* distribution from which the events in the figures have been drawn. **B.** Superposition of filtered signals (0.1–4 Hz) of scalp EEG recordings (FP1) corresponding to 300 slow waves showing fast depolarization (top) and 300 slow waves showing slow depolarization (bottom) of subject S1. The thick red line is the average profile of the shown filtered signals. The vertical dashed line marks the end of the depolarization (as has been defined in the text). The graphs in the right panels indicate the region in the *mode T_d_* distribution from which the events in the figure have been drawn.

**Figure 3:**
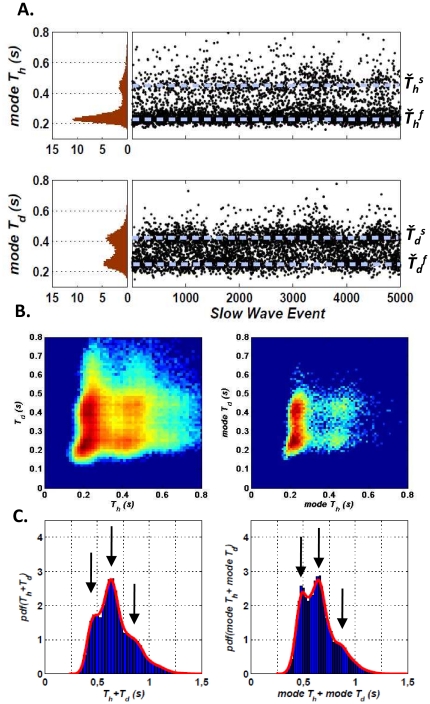
Distributions of mode T_h/d_ and study of the correlation between hyperpolarization and depolarization times. **A.** Mode T_h/d_ Probability Density Functions of a subject and mode T_h/d_ for each of the events detected in a whole night of sleep (5685 events were detected in that particular night of which only the first 5000 are shown). 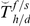 mark the position of the typical fast/slow hyperpolarization/depolarization mode times of the events of the subject. **B.** *Left:* T_d_ vs. T_h_ for a single subject. *Right:* mode T_d_ vs. mode T_h_ for a single subject. (A logarithmic scale has been used in both cases for the density color coding). **C.** Probability Density Function of (T_h_+T_d_) and (mode T_h_+mode T_d_). The continuous lines correspond to the estimated PDF using KDE (See Materials and Methods).

A group of 10 subjects with medically-refractory epilepsy were included in this study. Intracranial LFPs were recorded from the surface of the cortex (subdurally) or from depth electrodes implanted stereotactically in deeper cortical structures. Intracranial contacts sampled medial, lateral and basal frontal and temporal cortices, as well as medial and lateral parietal and occipital cortices (see Table S1 for further detail). All subjects had 2–3 selected overnight recordings. Slow waves were automatically identified from a single scalp electrode and only from segments of slow wave sleep using previous methodology [17] (see Materials and Methods section for further detail). Following previous works [6], [17], the onset of each wave was positioned at the maximum negativity of the waves, as this is considered to reflect the transition to the depolarizing phase of the intracellularly defined slow oscillation. We assumed that the first process, which leads up to the synchronization of populations of neurons in a silent hyperpolarization phase, starts at the first maximum before the central minimum of the slow oscillation (hyperpolarization onset) and finishes at the subsequent minimum (depolarization onset). We called T_h_ the time span of this phase [20] (see Figure 1A). At the depolarization onset, a second process starts where neurons are recruited to fire, leading to a global depolarization phase. We assumed that this phase ends at the first maximum after the onset of the depolarization and we called T_d_ its time span (Figure 1A). The analysis of intracranial recordings acquired simultaneously allowed us to directly characterize these different processes associated with scalp slow waves. In most cases, intracranial slow wave activities showed a reversed polarity with respect to the scalp EEG, confirming what has been previously shown in animal [1] and human studies [14]. Thus, the intracranial hyperpolarization phase was here considered to start at the first minimum and the depolarization onset was located at the maximum positivity peak (Figure 1A). We first analyzed the intracranial distributions for T_h_ and T_d_ measured over all detected slow waves (n = 33442) on all intracranial contacts (n = 417) (Figure 1B). For every recorded subject, we found a bimodal distribution for the depolarization and hyperpolarization time spans. We designate the characteristic times as 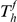 and 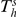 for the fast and slow hyperpolarization times and likewise 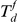, 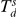 stand for the fast and slow depolarization times respectively (Figure 1C). The positions of the peaks, i.e. the values of the characteristic times, were remarkably consistent across all subjects (average (±s.d.) over all subjects of the positions of the peaks: 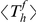 = 0.22±0.01 s, 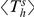 = 0.44±0.01 s, 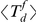 = 0.24±0.01 s, 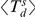 = 0.43±0.02 s) (Figure S1). Over multiple intracranial contacts, when analyzed one by one, we found exactly the same two peaks in the distributions of T_h_ and T_d_, suggesting that the bimodality cannot be simply explained by regional specificities of different cortical regions (Figure S2). As indicated by the visual inspection of several successive events (Figure 1B), the explanation of this bimodality lies in the co-existence of two different types of slow wave events (Figure S3). Examples of scalp EEG signals associated with fast/slow hyperpolarization/depolarization slow wave events are shown in Figure 2. To confirm this in a quantitative way, we determined the mode T_h_ and T_d_ of each event and obtained its statistical distribution over the whole night. For this purpose we used the Parzen's window method, also known as Kernel density estimation (henceforth referred to as KDE; see Materials and Methods). This method allows one to estimate the probability density function, and associated quantities such as the mode, of a certain variable given a finite (and possibly sparse) data sample. In the study of the distributions of modes, two peaks appeared, confirming that there is one slow (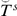) and one fast (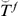) characteristic time for both the hyperpolarization and depolarization processes of a slow wave event: 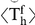 = 0.23±0.01 s, 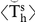 = 0.43±0.01 s, 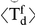 = 0.26±0.01 s, 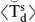 = 0.42±0.02 s (Figure 3A). Furthermore, all these characteristic times were homogenously identified over the course of an entire night of sleep (Figure 3A), suggesting that they were not reflecting state-dependent wave shapes (e.g. K-complexes of stage 2 and delta oscillations of stages 3–4) or the effect of the sleep pressure at the beginning of sleep [21]. However, complex laminar propagations of regional slow waves can implicate variable sources located along the cortical layers and may generate fluctuating wave polarities, depending on the spatial relationship between the intracranial electrode and the source of the oscillation [15]. These phase fluctuations could induce missing or double cycles in the characteristic times that may explain our bimodality. To examine this possibility, we characterized the cortical involvement in the fast/slow hyperpolarization/depolarization events by counting the total number of implicated intracranial contacts. As reported in Figure S4, we found no differences in the average number of contacts during fast and slow events, reflecting in both cases a similar involvement of a large number of cortical sites. This suggests that the bimodality and, in particular, the slow hyperpolarization/depolarization events are not a spurious effect of changing slow waves polarities due to complex propagation pathways of local slow waves. In order to further study whether slow and fast hyperpolarization/depolarization events reflect different physiological phenomena, we analyzed whether they were associated with differences in the power/amplitude of thalamically-generated spindles (8–18 Hz) (Figure S5). For 3 subjects with a good number of slow wave detections we observed that the amplitude of spindle activity after the slow wave minimum was significantly larger during slow-depolarization events, suggesting an implication of the thalamus in these events. Finally, the present results were obtained in individuals with epilepsy, who may have abnormal synchrony during seizure-free periods in their global activity patterns. In order to confirm the universality of the distributions of T_h_ and T_d_, scalp EEG recordings from 5 healthy subjects were analyzed. In every case, although the two modes were less clearly separated, a bimodal distribution was also observed with very similar peak values to those identified in intracranial data (Figure S6), confirming that epilepsy is unlikely to be the main source of the observed phenomenon.

We examined the existence of temporal relationships between consecutive fast and slow hyperpolarization or depolarization phases via a linear correlation analysis. We found a very weak correlation between T_h_ and T_d_ (mean correlation coefficient over all subjects: 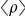 = 0.17±0.06, p<0.05) analysing the whole set of intracranial contacts, or between mode T_h_ and mode T_d_ (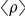 = 0.2±0.1, p<0.05) of the slow wave events. The weak correlation is clearly observed in Figure 3B where T_d_ is plotted versus T_h_ and mode T_d_ versus mode T_h_ for all the slow wave events of a representative subject. Even though fast hyperpolarization is more frequent, every possible combination of fast/slow hyperpolarization/depolarization can be identified in a slow wave event. In the same way, Figure 3C shows the distributions of the total time span T_h_+T_d_ and mode T_h_+mode T_d_. The weak correlation is again confirmed by the presence of three peaks representing all possible combinations between fast/slow hyperpolarization/depolarization events. In addition, we also examined the correlations over groups of temporally adjacent slow wave events. In this case we studied the correlation between the mode hyperpolarization times (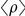 = 0.24±0.09, p<0.05), between the mode depolarization times 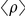 = 0.2±0.1, p<0.05) as well as the correlation between the depolarization time of one event and the hyperpolarization time of the following one (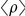 = 0.14±0.09, p<0.05). The observed weak linear correlation suggests that bursts of adjacent fast/slow hyperpolarization/depolarization waves appear in complex sequences during the same sleep stage.

### Hyperpolarization and depolarization propagation velocity

**Figure 4:**
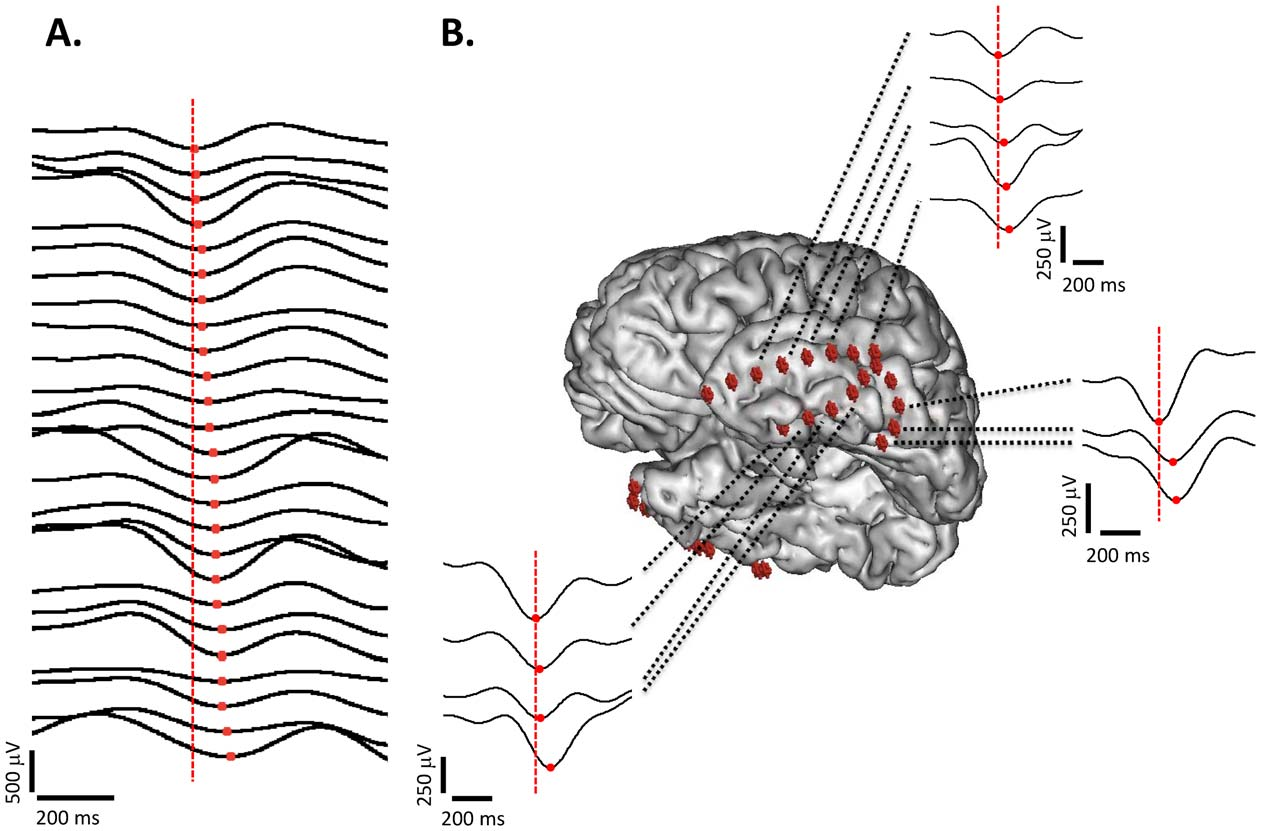
Propagation of the slow waves. **A.** Filtered (0.1–4 Hz) signals of intracranial EEG contacts of one global slow wave event ordered as a function of the position of the minimum (red dots). The vertical dashed line marks the position of the minimum of the first detection. **B.** Three examples of local propagation in the temporal lobe. Each example corresponds to a different slow wave event. In all cases the signals are linked to the position of the recording contacts in the brain, represented by red cubes.

Traditionally, the propagation of slow waves has been studied as the propagation of a depolarization wave (D-wave) [17]. We have indeed observed this propagation in our intracranial data. Figure 4 illustrates the propagation of a global slow wave event and three local propagation patterns. In this work we have also taken into account the hyperpolarization process that precedes the depolarization onset and considered the hyperpolarization as a traveling wave phenomenon (H-wave). The propagation velocity of both waves has been estimated with two methods. The first method consisted in the calculation of the instantaneous velocity of the slow wave. Given two consecutive detections of the same slow wave event, the speed was calculated from the distance between two contact locations, normalized to Talairach coordinate space [22], and the known time delay between the corresponding detections. This method assumes linear propagation of the waves and doesn't take into account particular characteristics of the cerebral cortex such as its convoluted surface. In order to reduce the error in the estimation of the speed we imposed a maximum distance threshold (40 mm) between the detection contacts. Histograms of the instantaneous speed were obtained for each subject and each type of wave. All histograms showed a clear mode peak, the exact value of which was estimated using KDE (an example can be seen in Figure S7). The mode velocity of the H-wave ranged from 0.35 to 0.56 m/s across subjects with a grand average of 0.5±0.1 m/s. For the D-wave, the mode velocity was inside the range 0.8–1.5 m/s and provided a grand average of 1.0±0.2 m/s. As we already mentioned, this method may provide underestimations of the traveling speeds due to the assumption of rectilinear propagation of the slow wave between the contacts. To further reduce the effect of these assumptions, we performed a second estimation. In this case we selected, for different subjects, pairs of close intracranial contacts (separation less than 15 mm) with a good percentage of detections. We then estimated the mean contact-to-contact velocity for each pair. A total number of 10 pairs of 7 different subjects were studied. The mean H-wave velocity ranged from 0.7 to 1.6 m/s with a grand average of 1.0±0.3 m/s. The D-wave mean speed ranged from 0.9 to 1.9 m/s with a grand average of 1.3±0.4 m/s. As expected, this second method provided larger estimates for the velocities. Using both estimations, we were not able to find any differences in the propagation velocities between fast- and slow-depolarization waves. A more refined description of their anatomical pathways is here needed to study subtle differences in their propagation speed.

### Origin and Propagation paths in networks of hubs

**Figure 5:**
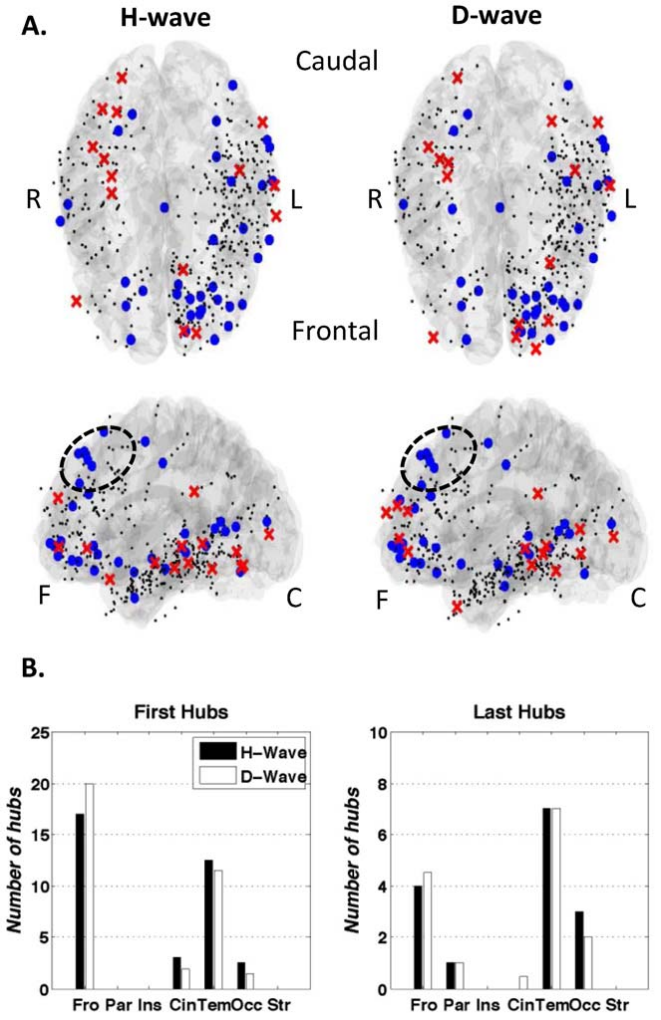
**Results** **of the propagation study.** **A.** *Left:* First and last hubs of the H-wave propagation of all studied subjects superimposed on the same reference brain (Top and left views). *Right:* First and last hubs of the D-wave propagation of all studied subjects superimposed on the same reference brain (Top and left views). The little black dots correspond to the implanted contacts not presenting a first/last hub character. These are shown to illustrate the total coverage of the sum of the individual implantations. Please note the final coverage of the sum of all the implantations is not homogeneous with 295 (71%) contacts is the left hemisphere and 122 (29%) in the right hemisphere. **B.** Number of first/last hubs found in each of the following regions: Frontal (Fro), Parietal (Par), Insula (Ins), Cingulate cortex (Cin), Temporal (Tem), Occipital (Occ) and Striatum (Str).

Close inspection of our data confirmed that the dynamics of the traveling slow wave are very far from the simple dynamics of a plane or spherical wave with a point-like and stable origin [19]. Each slow wave event seems to be unique in its origin and dynamics. Furthermore, our data confirmed that slow waves are not a global entrainment of all cortical regions via a single propagating wave but are often composed of complex local patterns with multiple points of origin. In order to deal with this complexity, we identified for each subject the set of intracranial contacts that were statistically more involved in all detected events, i.e. contacts with a number of detections of at least one standard deviation above the average. Defined in the network of slow wave propagations (the intracranial contacts acting as nodes and the links being causally related detections), these contacts can be seen as *hubs* since they would be the most connected nodes. We have studied the propagation by examining in which order each event visited these hubs. We have also calculated in which ordinal position (first, second, etc.) each hub was more likely to have been visited by analyzing the statistics over all the events. Finally, linking the *first* hubs with the *second* hubs, the *second* hubs with the *third* ones, etc., a directed graph could be constructed that established a causal relation between the hubs and hence a statistical propagation trend for each subject (Figure S8). In Figure 5A, the first (blue spheres) and last hubs (red crosses) of all subjects have been superimposed on a single cortical reconstruction. We have found no major differences for the propagation of H- and D-waves. For both the H-wave and the D-wave we found 35 first hubs and only 15 last hubs of a total of 417 contacts. In our opinion, this is a sign of the greater heterogeneity of the starting location of the slow waves. The majority of the first hubs were located in the anterior parts of the brain while the last hubs seemed to prefer the posterior parts. In particular, for the H-wave, we found 17 first hubs in the frontal lobe, 3 in the cingulate cortex, 12.5 in the temporal lobe and 2.5 in the occipital lobe (consistently with Table S1, contacts between two adjacent regions are considered as half in each one). The last hubs, on the contrary, were more present in posterior parts of the brain with 7 in the temporal lobe, 3 in the occipital region, 1 in the parietal lobe and only 4 situated in the frontal lobe. The results for the D-wave were similar with 20 of the 35 first hubs situated in the frontal lobe, 2 in the cingulate cortex, 11.5 in the temporal lobe and 1.5 in the occipital lobe. The last hubs of the D-wave were also more frequent in posterior regions with 7 in the temporal lob and 2 in the occipital cortex (the rest were located 4.5 in the frontal cortex, 1 in the parietal and 0.5 in the cingulate cortex) (Figure 5B). Even though the origin of the slow wave events could be very unordered, we identified a last-hubs-free region in the frontal cortex where first hubs tended to concentrate (F1/F2 cortex involving the superior frontal gyrus and the superior frontal sulcus). In Figure 5A a certain lateralization can be observed with more first hubs in the left hemisphere but this can be explained by the inhomogeneous distribution of the intracranial contacts with 295 (71%) located in the left hemisphere and only 122 (29%) implanted in the right hemisphere. Studies with a larger number of subjects or with a better spatial resolution are needed to confirm these results.

## Discussion

The present study explored the large-scale dynamics of sleep slow waves in the human cortex using intracranial EEG recordings. We report the following observations: 1) The hyperpolarization and depolarization processes have two distinct characteristic times. 2) A weak linear correlation could be identified between consecutive fast/slow hyperpolarization or depolarization phases. 3) Using two complementary methods, we estimated the average speed of both traveling waves as approximately 0.5±0.1 m/s (H-wave) and 1.0±0.2 m/s (D-wave) (1.0±0.3 m/s and 1.3±0.4 m/s, respectively). 4) For both waves, preferential starting locations were located in the anterior parts of the brain while they have a tendency to end in the posterior and temporal regions.

Aside from descriptive statistics of slow waves, the first new finding of this work was the determination of two characteristic times for both the hyperpolarization and depolarization processes. These slow and fast processes were remarkably consistent across all subjects and did not show significant correlation with regional specificities of different cortical regions or different sleep stages. A strong support of the physiological existence of this phenomenon is given by our analysis of scalp slow waves of healthy subjects that also exhibit a bimodality of hyperpolarization and depolarization times. This suggests that the bimodality is not related to the epileptic condition of our intracranially recorded subjects. Moreover, the demonstration of the bimodality from scalp EEG recordings allows us to discard the possibility that this phenomenon might be caused by fluctuating wave polarities around the recording intracranial sites [15]–[16] since the scalp electrodes are well over the cortical generators of slow waves. To further confirm this point we calculated the average number of contacts involved in the detection of fast and slow events. We found that both events involve a similar number of cortical sites, suggesting that the bimodality can not be explained by changing propagation pathways across the cortical layers giving rise to missing or double cycles in the recorded signal. Finally, we found a weak linear correlation between consecutive fast and slow hyperpolarization or depolarization phases, suggesting that these events appear in a mixed and complex way during the same sleep stage. We conjecture that nonlinear relations might exist between these two processes, particularly long-range temporal correlations at time scales of several minutes [23]. It is important to note that, in this study, the association of the up and down phases of the slow wave with the cellular hyperpolarization and depolarization processes is a hypothesis in nature since we have not correlated our data with intracellular recordings. As also assumed in other studies [17], this association is suggested by previous observations correlating cellular and EEG activity [1], [6]. However, further investigations at a cellular level in the human brain are necessary to validate these hypotheses [16].

The underlying causes of this novel bimodality of slow waves are still unclear. A possible explanation is that they could be related to the influences of subcortical regions on cortical synchrony. Indeed, although slow waves are generated within the cortex, as shown in animals with thalamic lesions [5] and confirmed in decorticated animals [24], recent data suggest a role of thalamic activity in triggering cortical slow waves through thalamocortical connections [8], [25], [26]. In particular, in cortical networks that lack thalamic input, i.e. cortical slabs [27] or slices [4], the hyperpolarization wave is considerably longer than if thalamic input is present. Furthermore, the mean duration of the depolarization phases increases with thalamic inputs [26], as also suggested by computational models [28]. Therefore, fast hyperpolarization or slow depolarization times could reflect the different implication of the thalamus. Preliminary analyses of the amplitude of spindle activity (8–18 Hz) around the slow wave minimum show differences in this frequency band between slow- and fast-depolarization slow wave events. In particular, for a small group of selected patients, we observed that slow depolarization events are accompanied by a strong and well-organized spindle activity (≈12–16 Hz) coinciding with cortical up-states (see Figure S5). On the contrary, fast depolarization events show a more diffuse spindle activity at cortical up-states but an alpha-like activity (≈8–10 Hz) can be seen coinciding with the depolarization onset. This mixture of activities related to fast-depolarization waves could suggest a similarity of these types of events with K-Complexes or micro-awaking episodes occurring during the active phase of the Cycle Alternating Patterns (CAP) [29]. In the CAP K-Complexes as well as other faster activities, including spindles, are present. In our opinion, these differences concerning spindle activity between slow and fast-depolarization waves reinforce the hypothesis of a distinct implication of the thalamus in these two different events. However, further work in this direction is needed to fully clarify the role of the thalamus in each type of slow wave processes.

Our second result is the direct estimation of the propagation speed of hyperpolarization and depolarization waves on the cortex. Using two different methods, we estimated the typical speed of both waves as 1 m/s. Interestingly, although this speed is of the same order of magnitude as previous estimates from EEG (2.7 m/s in [17]; 2.2 m/s in [19]) and computer simulations [30], the obtained values are somewhat smaller than previously published estimates of the depolarization propagation speed in humans. Linear interactions of voltages inherent in scalp EEG recordings [17] or their overlapping in source modeling [19] are most likely responsible for an overestimation of the intracranial propagation speed. However, our reported propagations were faster than the average speed around 0.1 m/s estimated in multi-site recordings of different cortical areas of anesthetized cats [31]. Our higher speeds may reflect not only a slight overestimation due to obliquely propagating waves or volume conduction [17], but also a higher speed of propagation in the human cortex secondary to its widespread long-range connectivity. Indeed, the speed of propagation in ferret cortical slices is low, around 0.01 m/s, given that it relies exclusively on local connectivity [4].

Our data were recorded in medicated epilepsy patients in whom the propagation of slow waves may be affected by epileptic disturbances. Nevertheless, as several other sleep studies using intracranial data [15], [16], we believe that the effects of pathology on our results are minor for the following reasons. First, high epileptic activities were visually identified and removed from our data. Second, as imposed by our automatic detection, slow wave dynamics were within the expected normal range, including standard morphologies, durations, amplitudes and spatial extent. Third, from the total number of intracranial contacts, 83% were located outside the epileptogenic zone. Finally, interictal epileptic events have markedly different temporal dynamics with an extremely fast synchronization but more limited in their spatial extent. The propagations we describe here showed remarkable global dynamics, reproducible from one patient to another.

In addition, there are clear parallels between the results of [19] and our data. In both studies, spontaneous slow waves were reported to propagate across the cortical area. Nevertheless, a high spatial variability of the propagation was found. In particular, slow waves were composed of multiple propagation paths which can vary from one wave to another and with several points of origin [18]. This variable network of propagations may reflect the level of activity-dependent synchronization achieved in cortical neural ensembles, which depends on the local homeostatically regulated average synaptic strength [32]. In our work, we have chosen to reduce this complexity by only studying the preferential trajectories between intracranial sites statistically more involved in slow waves. We found that, for both hyperpolarization and depolarization waves, the majority of the first hubs were localized in the anterior parts of the brain while the last hubs seemed to prefer the posterior parts. This further confirms the presence of cortical generators in the frontal cortex with enhanced intrinsic excitability, which could consistently generate slow waves during sleep.

## Materials and Methods

### Ethics Statement

The EEG signals were recorded for the presurgical evaluation of the patients. Patients gave a written consent for a research use of these data. The procedure was approved by our local Ethical Committee (CPP Ile-de France VI, Groupe Hospitalier Pitié-Salpêtrière).

### Subjects and EEG acquisition

For the intracranial study, the sleep EEG recordings of 10 subjects (5 Females; age range, 18–49; Mean Age = 32,9) with refractory partial epilepsy undergoing presurgical evaluation, hospitalized between February 2002 and July 2007 in the epilepsy unit at the Pitié-Salpêtrière hospital in Paris, were analyzed. Each patient was continuously explored during several days with intracranial and scalp electrodes (Nicolet acquisition system, CA, US). Depth electrodes were composed of 4 to 10 cylindrical contacts 2.3 mm long, 1 mm in diameter, 10 mm apart center-to-center, mounted on a 1 mm wide flexible plastic probe. Subdural electrodes were strips with 4 to 8 one-sided circular contacts, 2.3 mm in diameter and with a center-to-center separation of 10 mm. The effective surface area was 7.2 mm^2^ for depth contacts and 4.15 mm^2^ for subdural contacts. Pre and post implantation MRI scans were evaluated to anatomically and precisely locate each contact along the electrode trajectory. Talairach coordinates of intracranial contacts were estimated with the BrainVisa/Anatomist software package (http://brainvisa.info/). The placement of electrodes within each patient was determined solely by clinical criteria; however, the routine clinical use of broad anatomical coverage for intracranial recordings provided a large sample of electrophysiological data from tissue outside of the epileptogenic zone. In particular, a total number of 417 intracranial contacts were analyzed from which 83% were located outside the epileptogenic zone. The intracranial contacts were distributed in the different brain regions in the following way: 122 in Frontal regions, 12 in Parietal regions, 19 in Occipital regions, 231.5 in Temporal regions, 23 in the Cingulate cortex, 8 in the Insula and 1.5 in the Striatum (contacts between two adjacent regions were considered as half in each one). Signals were digitalized at 400 Hz. Common average reference montage was chosen to normalize the signals. Noisy electrodes and those presenting high epileptic activity were excluded from the average by visual inspection. A data set of 22 complete nights of sleep with at least two complete sleep cycles was obtained with each subject contributing with 2–3 nights. Each night was scored for sleep stages using the software Somnologica™. All patients gave their informed consent and procedures were approved by the local ethical committee (CCP).

Scalp sleep EEG recordings of 5 male healthy subjects were also analyzed. For each subject, 3 complete nights were studied. Electrographic (EEG), electro-oculogram (EOG) and chin electro-myogram (EMG) activity were concurrently monitored and used to classify the different sleep states.

Digital bandpass filtering between frequencies f_1_–f_2_ was, in all cases, implemented through a forward-backward digital infinite impulse response (IIR) type II Chebyshev filter (passband: f_1_≤f≤f_2_ Hz, attenuation ≤1 dB, monotonic; stopband: f≤(f_1_−k) or f≥(f_2_+k) Hz, where k = 0.5 for f_1,2_≥1 Hz or k = 0.05 otherwise, attenuation ≥100 dB, equiripple). Electrical line noise at 50 Hz was suppressed by a bandstop filter of the same type. All analyses were implemented in MATLAB®(The MathWorks™, MA, USA).

### Sleep Oscillations detection criteria

For the intracranial-EEG subjects, the Slow Oscillations were first detected from a single scalp electrode and only from segments of NREM sleep. Since not all subjects shared exactly the same number of scalp electrodes, FP1 was chosen for the analysis because it was systematically recorded and because it had been previously used for studying slow wave activity [21]. For the automatic detection of the slow waves, all signals were bandpass-filtered between 0.1–4 Hz. The criteria for the automatic detection in the scalp EEG were chosen following those in the literature [17], [19]: (1) separation between the negative zero crossing and the subsequent positive zero crossing of 0.125–1.0 s; (2) separation between the positive zero crossing and the following negative zero crossing of 0.125–1.5 s; (3) negative peak between the first two zero crossings with a voltage V
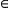
[−400,−80] µV; (4) a negative to positive peak-to-peak amplitude >140 µV. We disregarded the cases presenting more than one minimum between the crossings. Once a slow oscillation was detected in the Scalp EEG, a correlation study was performed to determine which other intracranial contacts had detected that particular slow wave as well. We opened a 1.5 s window around the scalp minimum and we calculated the correlation coefficient between the scalp signal and the intracranial contacts signal. We assume an intracranial electrode detected the slow oscillation if the absolute value of the correlation coefficient is >0.6 and the amplitude of the minimum (maximum in the case of inversion) has a magnitude at least 25% of that in scalp. We identified a slow wave event when at least 40% of the active contacts detected the slow oscillation.

For the scalp-EEG subjects, the Slow Oscillations were first detected from the C3 electrode. The criteria for the detection were the same as in the intracranial case. Similarly to the intracranial case, a correlation study was performed to determine which scalp contacts had also detected the slow wave event.

### Kernel density estimation (KDE)

Kernel density estimation is a method for the estimation of probability density functions and modes [33].

The theoretical basis of the KDE method is the following: Given a set of i.i.d. random variables X_1_,X_2_,X_3_… we can estimate the corresponding probability density function as
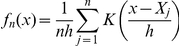
where *K(…)* is called the kernel and *h* the bandwidth. The kernel is usually chosen to be of a Gaussian type
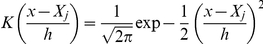
We have implemented this method using the function ksdensity of the software package MATLAB®(The MathWorks™, MA, USA).

## Supporting Information

Figure S1 **Examples of T_h_/T_d_ and mode T_h_/mode T_d_ distributions.** Distributions of T_h_/T_d_ and mode T_h_/mode T_d_ for 6 subjects (S2 to S7) with intracranial electrode implantations (See Table S1 for further detail).
(TIF)

Figure S2 **Statistics of T_h_ and T_d_ for single intracranial contacts.** Histograms of Th and Td for four different recording contacts. The first one is situated in the frontal superior cortex, the second in the temporal T1 cortex, the third in the parietal cortex close to the lateral sulcus and the fourth in the occipital O2 cortex. The frontal and temporal contacts correspond to subject S1 (see Table S1), the parietal contact to subject S5 and the occipital contact to subject S4.
(TIFF)

Figure S3 **Examples of SW events with different characteristic hyperpolarization and depolarization times.** **A.** *Left:* example of a characteristic fast hyperpolarization event. *Right:* Example of a characteristic slow hyperpolarization event. The position of the mode T_h_ of the events in the probability distribution has been marked in the upper graph. **B.** *Left:* Example of a characteristic slow depolarization event. *Right:* Example of a characteristic fast depolarization event. The position of the mode T_d_ of the events in the probability distribution has been marked in the upper graph.
(TIF)

Figure S4 **Average number of intracranial contacts involved in the detection of fast/slow hyperpolarization/depolarization events.** The averages shown were calculated for eight subjects (S1 to S8 in Table S1) for their fast/slow hyperpolarization (upper panel) and depolarization (lower panel) events.
(TIF)

Figure S5 **Analysis of thalamically-generated spindles during fast and slow depolarization events.** Each panel (from 1 to 5) corresponds to one contact of an intracranial electrode (blue dots in the anatomical figure). Fast and slow depolarization events are studied separately. In each panel from top to bottom: Filtered signals (0.1–4 Hz) of the detected slow waves aligned around their central peak (red) with the average RMS activity (green line) in the band 8–18 Hz; Average of their time-frequency representations.
(TIFF)

Figure S6 **Examples of T_h_ and T_d_ distributions for three healthy patients.** These distributions have been obtained from the analysis of 3 nights of sleep scalp-EEG recordings for each patient.
(TIF)

Figure S7 **Example of the estimation of the speed of propagation.** Histograms of the estimated speed (calculated as described in the text) for the H-wave and D-wave of subject S2. The mode peak is clearly visible.
(TIF)

Figure S8 **Illustration of the method for the study of the propagation.** *Top left*: Percentage of the total number of events of a subject (S5) detected by each of its intracranial contacts. The dashed line corresponds to the threshold of one standard deviation. *Top right*: Detection probability vs. order of detection for each hub. This probability has been calculated over all the events of the subject. The black circles point the position of maximum detection probability of each hub. *Bottom*: Reconstruction of the directed graph on the intracranial implantation (temporal implantation in this case) and a schematic representation of the graph construction procedure. The blue spheres mark the hubs that preferentially perform the first detection and the red crosses mark the hubs that preferentially perform the last ones.
(TIF)

Table S1 **Details of the intracranial implantations.** Contacts in the epileptic ictal zone correspond here to contacts associated to seizure onsets. Contacts in the epileptic interictal zone correspond here to contacts associated to epileptic spikes during interictal periods. Contacts between two adjacent regions were considered as half in each one. Legend: Fro (Frontal), Par (Parietal), Occ (Occipital), Cin (Cingulate), Ins (Insula), Str (Striatum).
(DOC)
