# Supplementary figures and images for: Large-Scale Cortical Dynamics of Sleep Slow Waves
Source: PLoS One. 2012 Feb 17;7(2):e30757. doi: 10.1371/journal.pone.0030757 (PMC3281874; doi:10.1371/journal.pone.0030757)

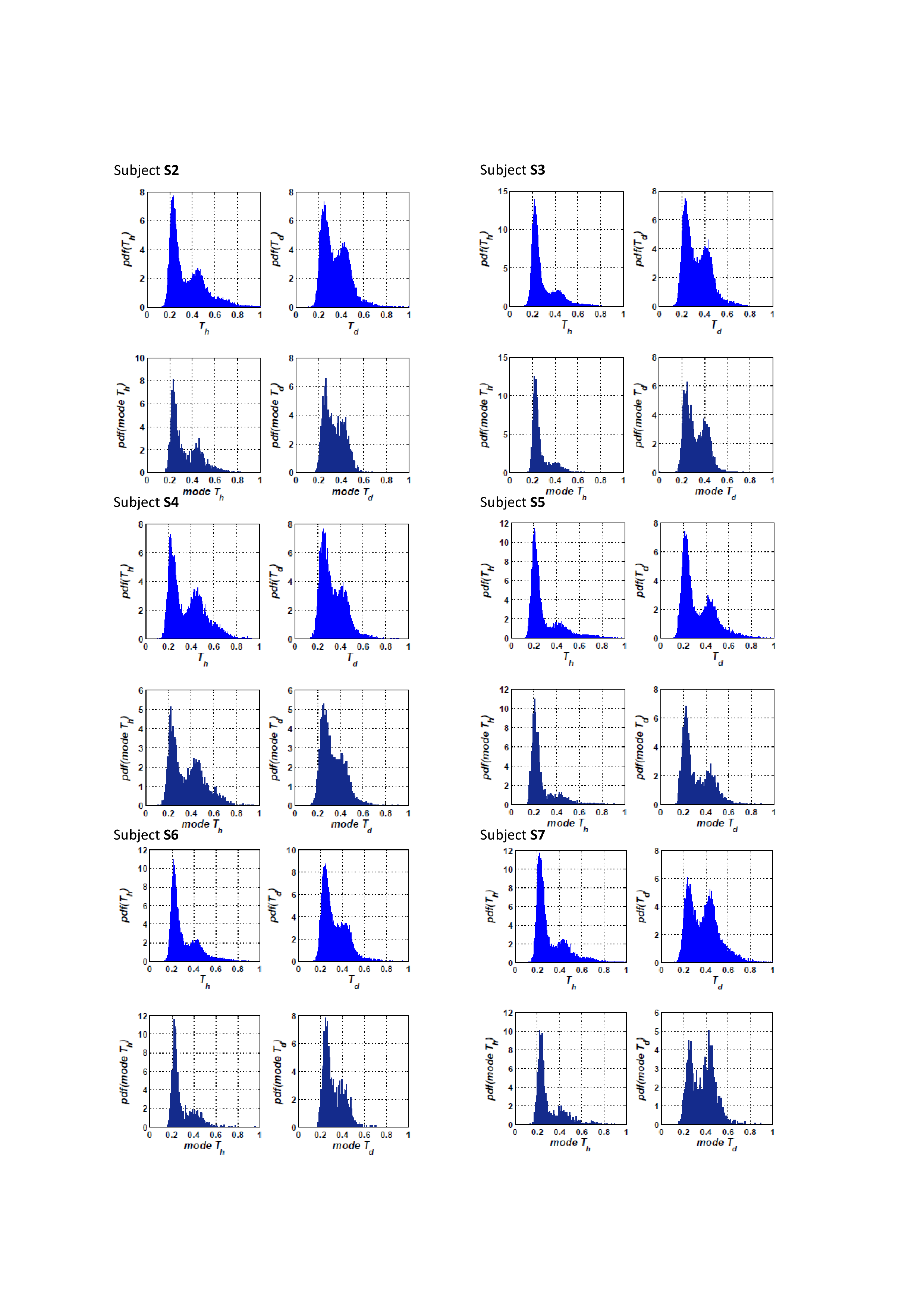

Supplement: Figure S1 — Examples of Th/Td and mode Th/mode Td distributions. Distributions of Th/Td and mode Th/mode Td for 6 subjects (S2 to S7) with intracranial electrode implantations (See Table S1 for further detail). (TIF) [file pone.0030757.s001.tif]

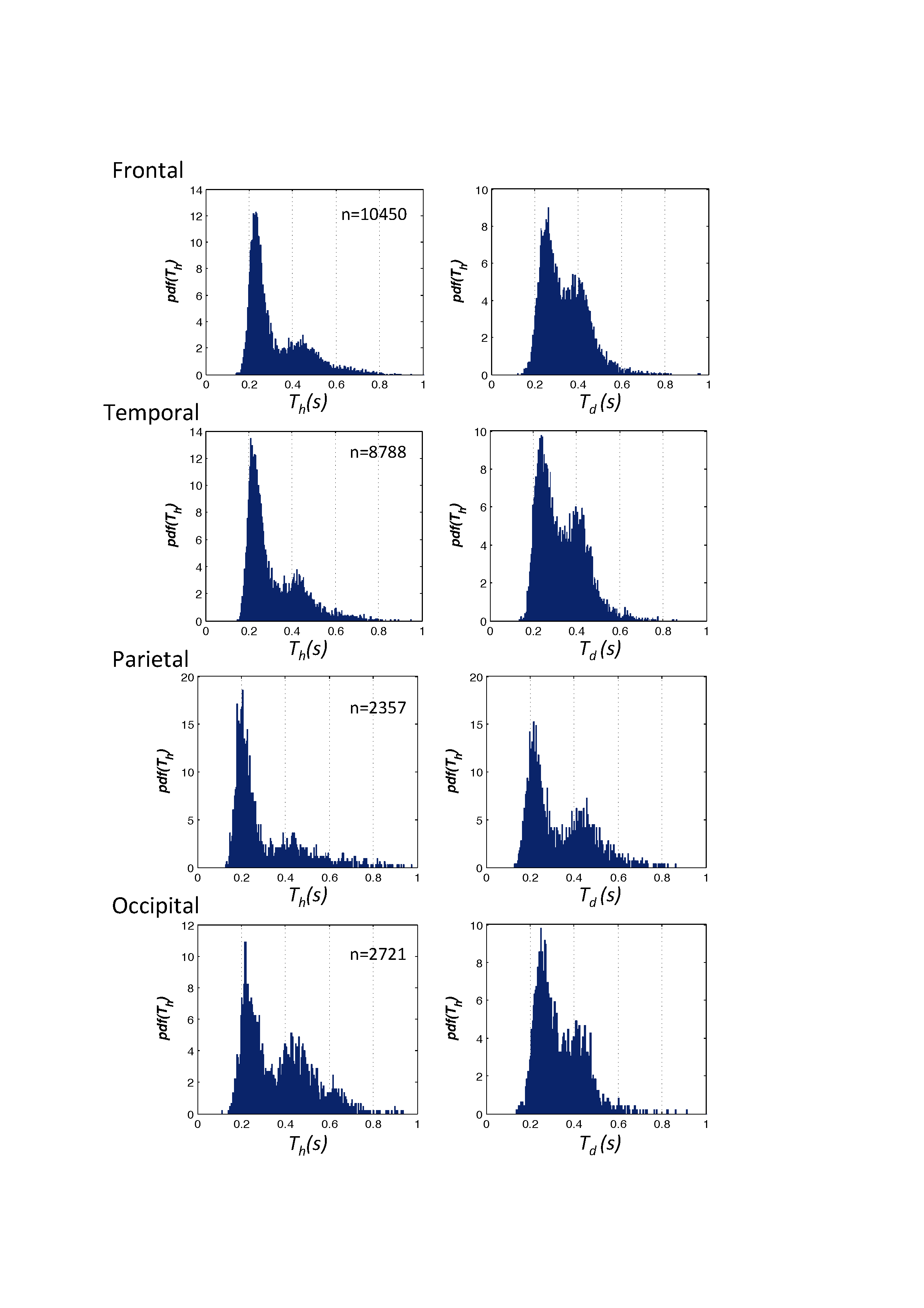

Supplement: Figure S2 — Statistics of Th and Td for single intracranial contacts. Histograms of Th and Td for four different recording contacts. The first one is situated in the frontal superior cortex, the second in the temporal T1 cortex, the third in the parietal cortex close to the lateral sulcus and the fourth in the occipital O2 cortex. The frontal and temporal contacts correspond to subject S1 (see Table S1), the parietal contact to subject S5 and the occipital contact to subject S4. (TIFF) [file pone.0030757.s002.tif]

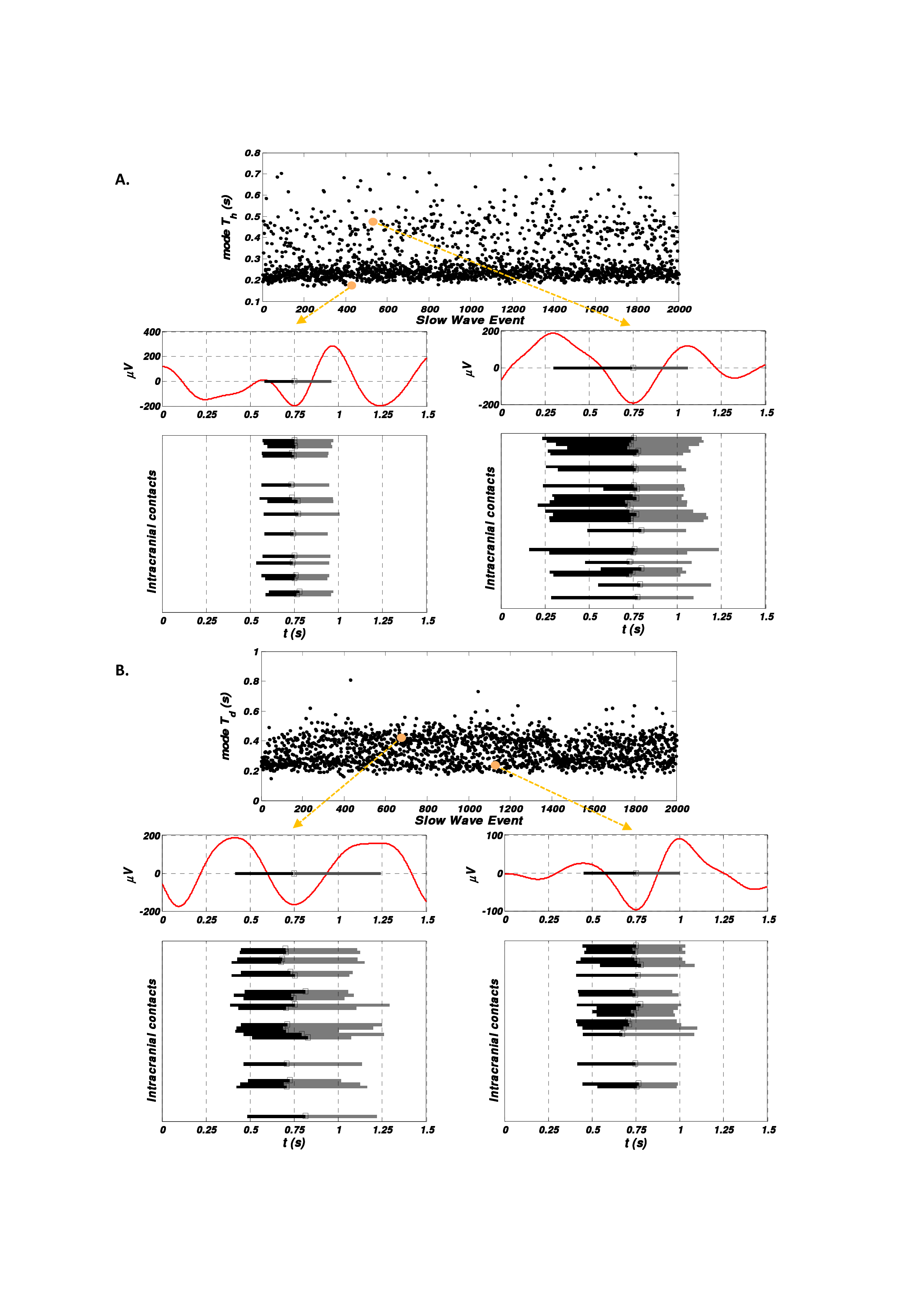

Supplement: Figure S3 — Examples of SW events with different characteristic hyperpolarization and depolarization times. A. Left: example of a characteristic fast hyperpolarization event. Right: Example of a characteristic slow hyperpolarization event. The position of the mode Th of the events in the probability distribution has been marked in the upper graph. B. Left: Example of a characteristic slow depolarization event. Right: Example of a characteristic fast depolarization event. The position of the mode Td of the events in the probability distribution has been marked in the upper graph. (TIF) [file pone.0030757.s003.tif]

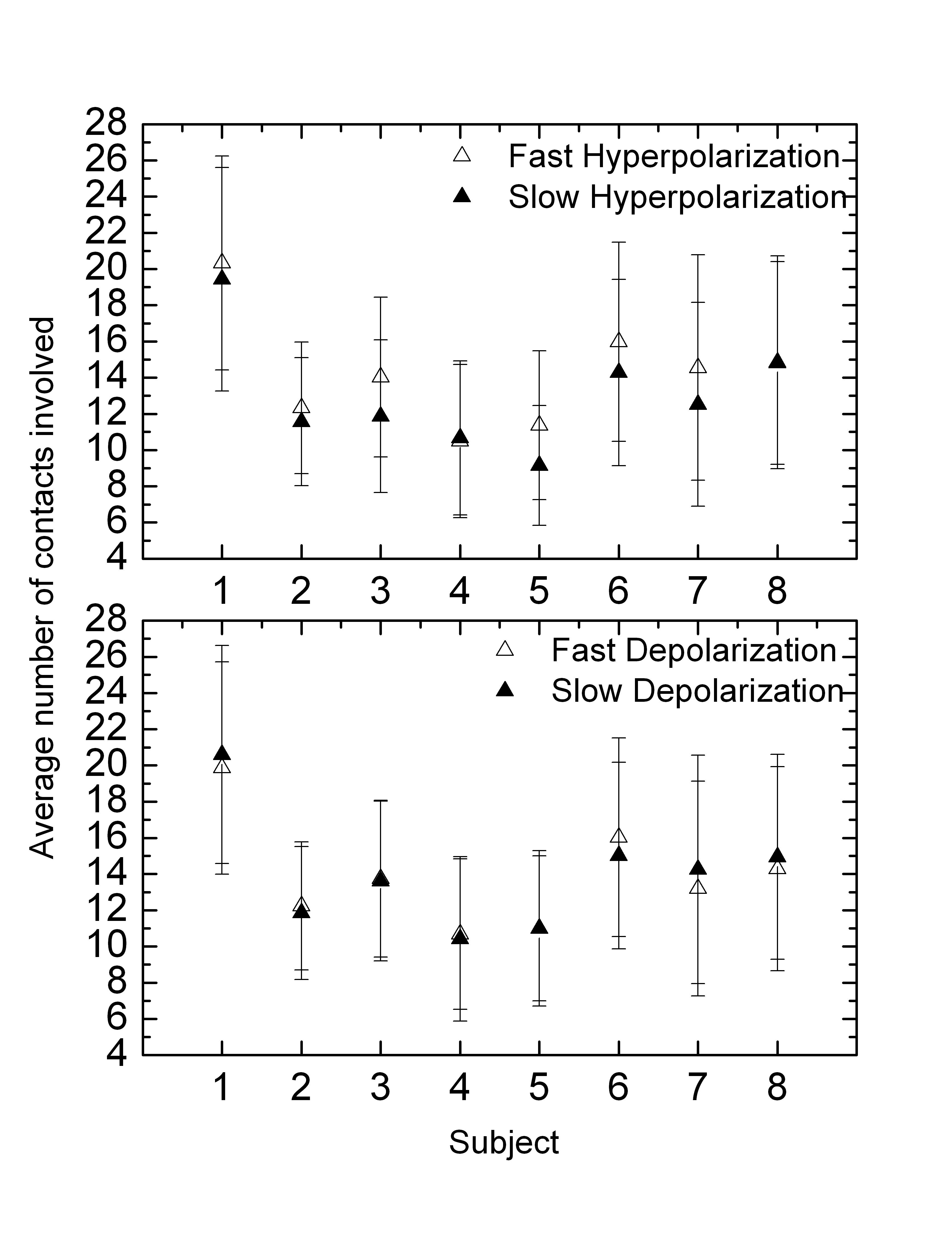

Supplement: Figure S4 — Average number of intracranial contacts involved in the detection of fast/slow hyperpolarization/depolarization events. The averages shown were calculated for eight subjects (S1 to S8 in Table S1) for their fast/slow hyperpolarization (upper panel) and depolarization (lower panel) events. (TIF) [file pone.0030757.s004.tif]

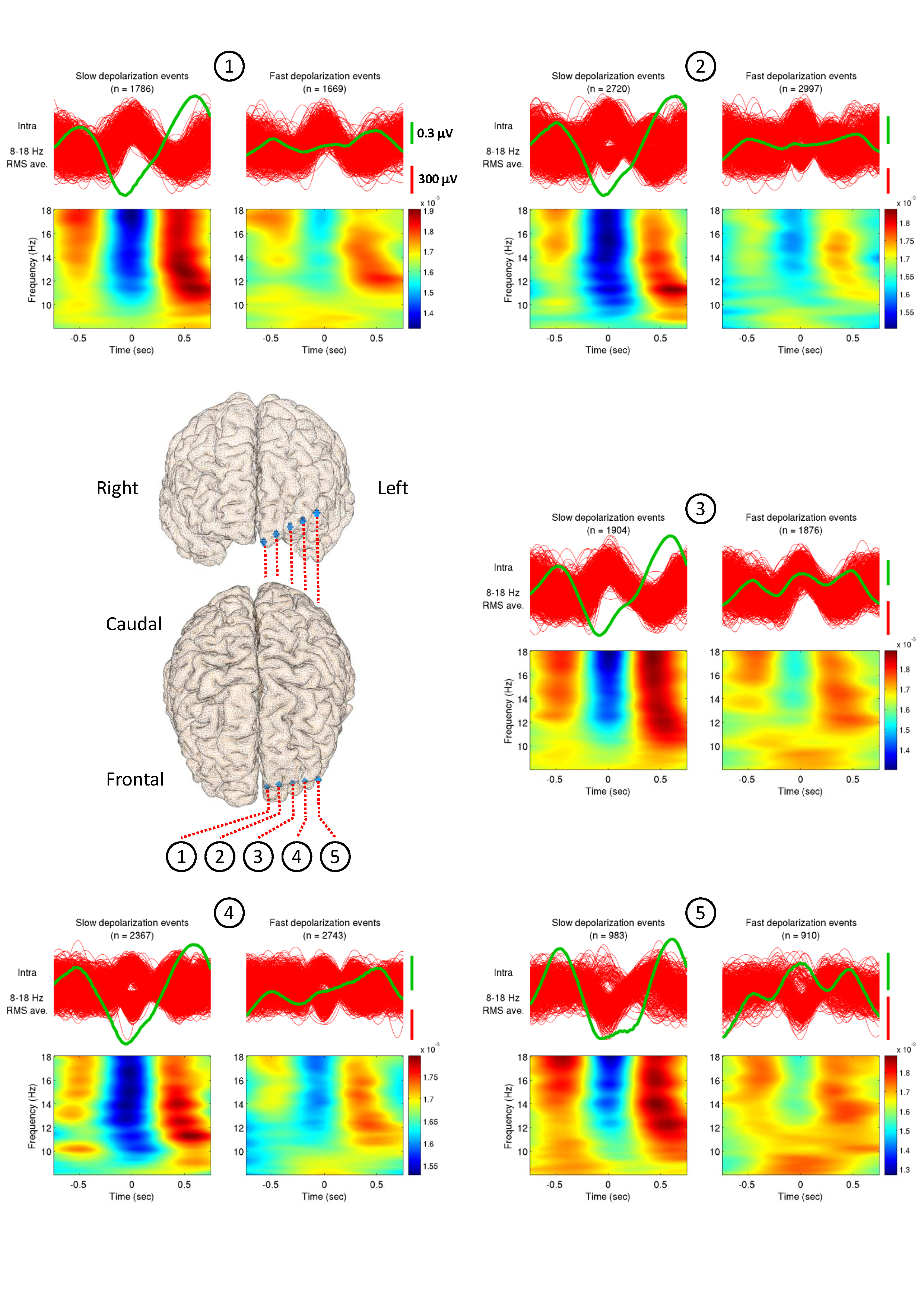

Supplement: Figure S5 — Analysis of thalamically-generated spindles during fast and slow depolarization events. Each panel (from 1 to 5) corresponds to one contact of an intracranial electrode (blue dots in the anatomical figure). Fast and slow depolarization events are studied separately. In each panel from top to bottom: Filtered signals (0.1–4 Hz) of the detected slow waves aligned around their central peak (red) with the average RMS activity (green line) in the band 8–18 Hz; Average of their time-frequency representations. (TIFF) [file pone.0030757.s005.tif]

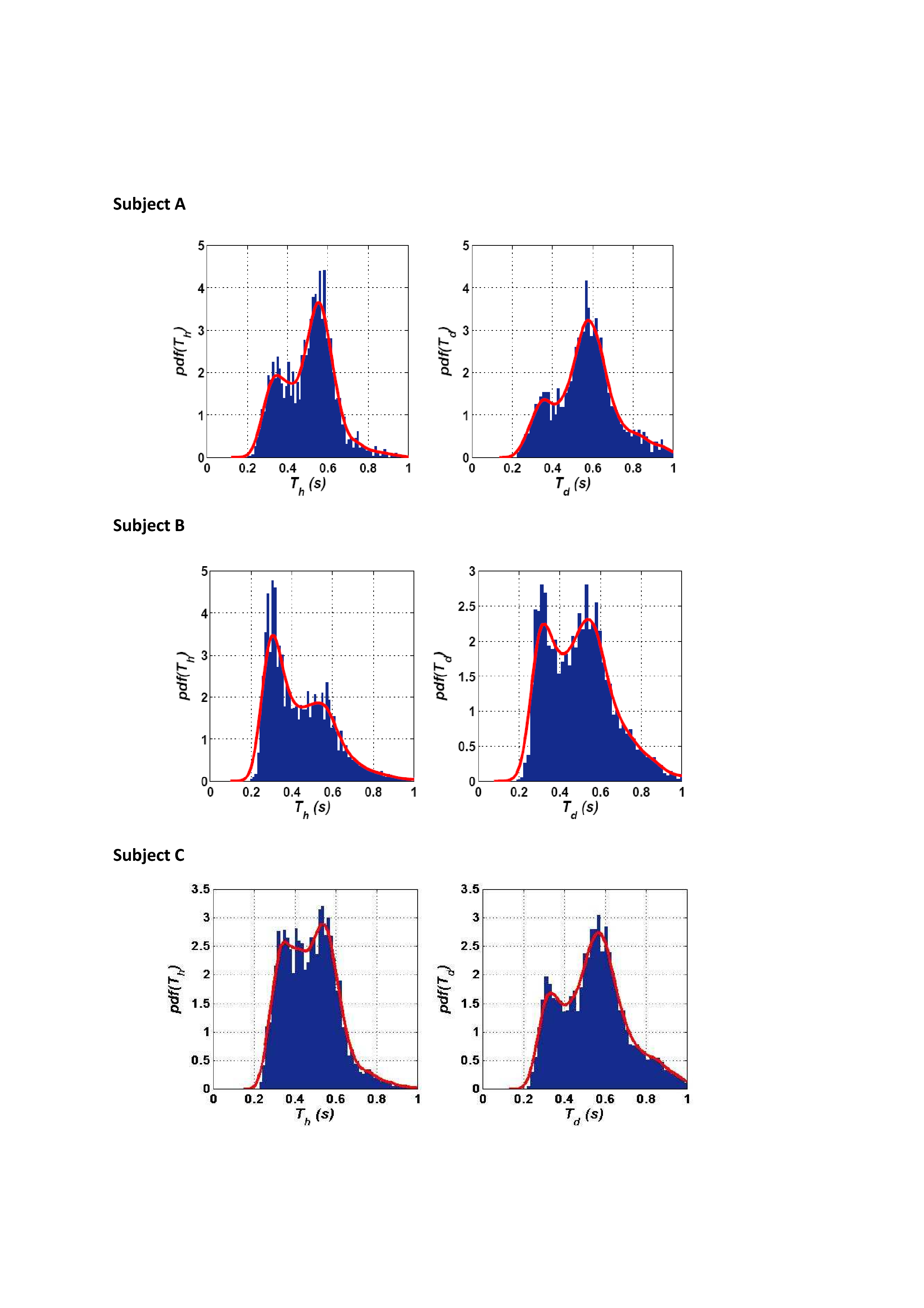

Supplement: Figure S6 — Examples of Th and Td distributions for three healthy patients. These distributions have been obtained from the analysis of 3 nights of sleep scalp-EEG recordings for each patient. (TIF) [file pone.0030757.s006.tif]

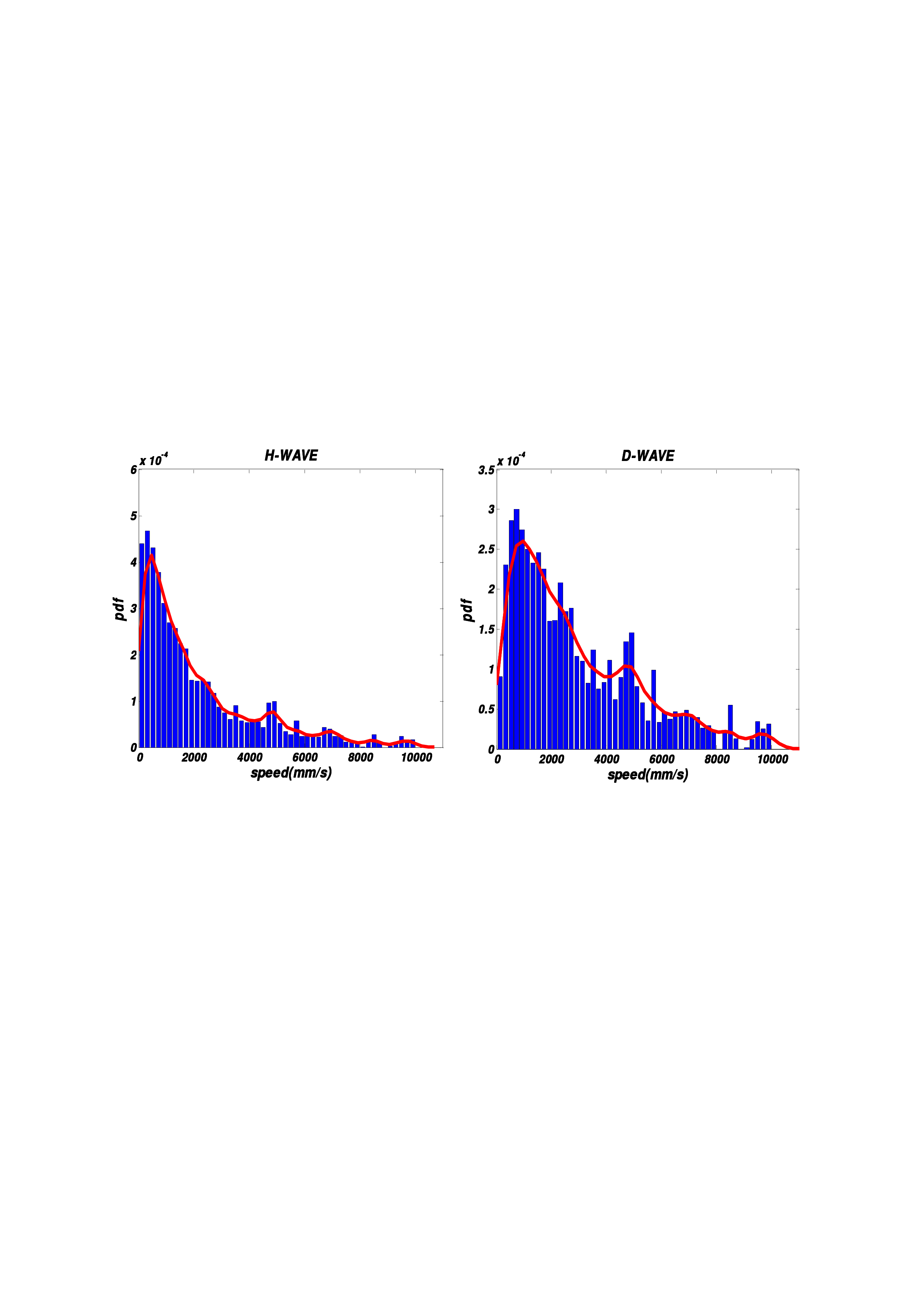

Supplement: Figure S7 — Example of the estimation of the speed of propagation. Histograms of the estimated speed (calculated as described in the text) for the H-wave and D-wave of subject S2. The mode peak is clearly visible. (TIF) [file pone.0030757.s007.tif]

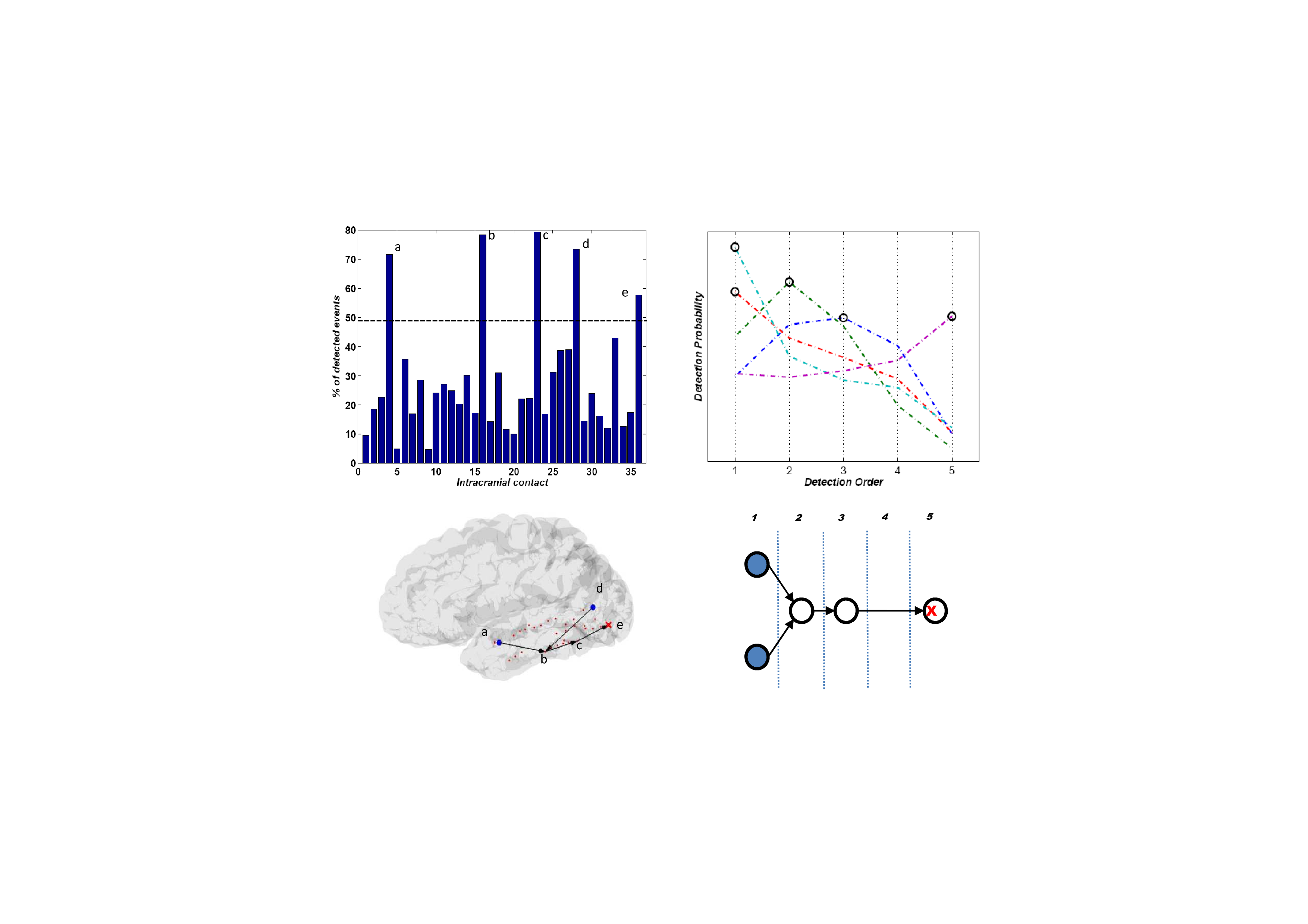

Supplement: Figure S8 — Illustration of the method for the study of the propagation. Top left: Percentage of the total number of events of a subject (S5) detected by each of its intracranial contacts. The dashed line corresponds to the threshold of one standard deviation. Top right: Detection probability vs. order of detection for each hub. This probability has been calculated over all the events of the subject. The black circles point the position of maximum detection probability of each hub. Bottom: Reconstruction of the directed graph on the intracranial implantation (temporal implantation in this case) and a schematic representation of the graph construction procedure. The blue spheres mark the hubs that preferentially perform the first detection and the red crosses mark the hubs that preferentially perform the last ones. (TIF) [file pone.0030757.s008.tif]
