# Supplementary material for: Large-Scale Cortical Dynamics of Sleep Slow Waves
Source: PLoS One. 2012 Feb 17;7(2):e30757. doi: 10.1371/journal.pone.0030757 (PMC3281874; doi:10.1371/journal.pone.0030757)
Supplement: Table S1 — Details of the intracranial implantations. Contacts in the epileptic ictal zone correspond here to contacts associated to seizure onsets. Contacts in the epileptic interictal zone correspond here to contacts associated to epileptic spikes during interictal periods. Contacts between two adjacent regions were considered as half in each one. Legend: Fro (Frontal), Par (Parietal), Occ (Occipital), Cin (Cingulate), Ins (Insula), Str (Striatum). (DOC) [file pone.0030757.s009.doc]

| Subject |  | **Number of intracranial contacts per cortex region** | | | | | | | | | | | | | | | | | | | | | | |
| --- | --- | --- | --- | --- | --- | --- | --- | --- | --- | --- | --- | --- | --- | --- | --- | --- | --- | --- | --- | --- | --- | --- | --- | --- |
|  |  | *Total implanted contacts* | | | | | | |  | *Contacts in the epileptic ictal zone* | | | | | | |  | *Contacts in the epileptic interictal zone* | | | | | | |
|  |  | **Fro** | **Par** | **Occ** | **Tem** | **Cin** | **Ins** | **Str** |  | **Fro** | **Par** | **Occ** | **Tem** | **Cin** | **Ins** | **Str** |  | **Fro** | **Par** | **Occ** | **Tem** | **Cin** | **Ins** | **Str** |
| *S1* |  | 18 | 0 | 0 | 25 | 9 | 1,5 | 1,5 |  | 2 | 0 | 0 | 2 | 4 | 1 | 1 |  | 3 | 0 | 0 | 10 | 0 | 0 | 0 |
| *S2* |  | 27 | 0 | 0 | 0 | 2 | 2 | 0 |  | 3 | 0 | 0 | 0 | 0 | 0 | 0 |  | 3 | 0 | 0 | 0 | 0 | 0 | 0 |
| *S3* |  | 24,5 | 0 | 0 | 13 | 2,5 | 1 | 0 |  | 6 | 0 | 0 | 1 | 0 | 1 | 0 |  | 6 | 0 | 0 | 11 | 0 | 0 | 0 |
| *S4* |  | 0 | 4,5 | 4,5 | 17 | 0 | 0 | 0 |  | 0 | 4,5 | 1,5 | 2 | 0 | 0 | 0 |  | 0 | 2 | 2,5 | 8,5 | 0 | 0 | 0 |
| *S5* |  | 17 | 6 | 0 | 3 | 2 | 3 | 0 |  | 0 | 0 | 0 | 2 | 0 | 1 | 0 |  | 3 | 0 | 0 | 0 | 0 | 2 | 0 |
| *S6* |  | 17,5 | 0 | 7 | 16 | 4 | 0,5 | 0 |  | 8,5 | 0 | 0 | 0 | 3,5 | 0 | 0 |  | 4 | 0 | 1 | 2 | 0 | 0 | 0 |
| *S7* |  | 0 | 1,5 | 0 | 48 | 0,5 | 0 | 0 |  | 0 | 0 | 0 | 5 | 0 | 0 | 0 |  | 0 | 0 | 0 | 17,5 | 0,5 | 0 | 0 |
| *S8* |  | 0 | 0 | 7,5 | 48,5 | 0 | 0 | 0 |  | 0 | 0 | 0 | 7 | 0 | 0 | 0 |  | 0 | 0 | 0 | 24 | 0 | 0 | 0 |
| *S9* |  | 0 | 0 | 0 | 36 | 0 | 0 | 0 |  | 0 | 0 | 0 | 8 | 0 | 0 | 0 |  | 0 | 0 | 0 | 10 | 0 | 0 | 0 |
| *S10* |  | 18 | 0 | 0 | 25 | 3 | 0 | 0 |  | 5 | 0 | 0 | 0 | 1 | 0 | 0 |  | 0,5 | 0 | 0 | 10 | 0,5 | 0 | 0 |
| *Total* |  | *122* | *12* | *19* | *232* | *23* | *8* | *1,5* |  | *24,5* | *4,5* | *1,5* | *27* | *8,5* | *3* | *1* |  | *19,5* | *2* | *3,5* | *93* | *1* | *2* | *0* |
